# Versatile and efficient chromatin pull-down methodology based on DNA triple helix formation

**DOI:** 10.1038/s41598-018-24417-9

**Published:** 2018-04-12

**Authors:** Asako Isogawa, Robert P. Fuchs, Shingo Fujii

**Affiliations:** 1 DNA Damage Tolerance CNRS, UMR7258, Marseille, F-13009 France; 2 Inserm, U1068, CRCM, Marseille, F-13009 France; 3 Institut Paoli-Calmettes, Marseille, F-13009 France; 4 Aix-Marseille University, UM 105, F-13284 Marseille, France; 5 Present Address: Harvard Medical School, Boston, MA 02115 USA

## Abstract

The goal of present paper is to develop a reliable DNA-based method for isolation of protein complexes bound to DNA (Isolation of DNA Associated Proteins: IDAP). We describe a robust and versatile procedure to pull-down chromatinized DNA sequences-of-interest by formation of a triple helix between a sequence tag present in the DNA and a complementary triple helix forming oligonucleotide (TFO) coupled to a desthiobiotin residue. Following optimization to insure efficient recovery of native plasmids via TFO probe *in vitro*, the procedure is shown to work under various experimental situations. For instance, it allows capture proteins associated to plasmids hosted in *E. coli*, and is also successfully applied to recovering nucleosomes *in vitro* opening many possibilities to study post translational modifications of histones in a genuine nucleosome context. Incubation in human nuclear extracts of a plasmid carrying a NF-κB model promoter is shown to pull-down a specific transcription factor. Finally, isolation of a specific locus from human genomic chromatin has been successfully achieved (Chromatin-of-Interest Fragment Isolation: CoIFI). In conclusion, the methodology can be implemented for capturing proteins that specifically bind to any sequence-of-interest, DNA adduct or secondary structure provided a short sequence tag for triple helix formation is located nearby.

## Introduction

Characterization of the proteins involved in various DNA transactions (e.g., replication, repair, transcription, nucleosome dynamics) is an essential requirement for the proper understanding of how protein networks control gene expression in fundamental as well as in clinical areas. For this purpose, numerous *in vitro* and *in vivo* experimental approaches have been developed. Typical *in vitro* examples use synthetic oligonucleotides containing a DNA sequence-of-interest mixed with purified or crude proteins, followed by analysing proteins bound on the DNA^1,2^. In contrast, analysing proteins bound to specific DNA sequences *in vivo* is way more challenging due to intrinsic cellular complexities. Several methodologies have been reported for isolating protein/DNA complexes from living cells. One way is to implement immunoprecipitation-based methods^3–6^ in which protein complexes that contain a protein of interest are isolated as in regular chromatin immunoprecipitation (ChIP) methods. The associated proteins instead of DNA are analysed. However, these approaches do not ensure that all the isolated proteins are indeed bound to DNA. A more direct way consists of isolating DNA/protein complexes by affinity capture of modified or intact DNA^7–11^. These DNA-based methods ensure, in principle, that the isolated proteins are bound to DNA either directly or as part of a complex.

Triple helix (triplex) formation of nucleic acids (RNA triplex) was initially reported in 1957^12^. Nearly 30 years later, formation of DNA triplex was also reported^13^. Since then, the properties of triplexes have been intensively studied because they potentially offer attractive applications for engineering and therapeutic purposes^14–16^. The key feature of triplexes is that a third strand (referred to as Triplex Forming Oligonucleotide (TFO)) consisting of either a pyrimidine-rich or a purine-rich sequence, forms Hoogsteen (or reverse Hoogsteen) base pairs with a complementary purine-rich strand in the major groove of double-stranded DNA (dsDNA) without disrupting its canonical Watson-Crick base pairs. While biotinylated TFO probes with normal deoxyribonucleotides were shown to specifically capture dsDNA *in vitro*^17^, numerous efforts were deployed to further increase the stability of TFO-mediated triplexes. These improvements involved both chemically altered nucleotides (e.g., peptide nucleic acid (PNA), locked nucleic acid (LNA))^18,19^ and the introduction of DNA intercalators (e.g., acridine, psoralen) linked to an extremity of the TFO molecule^20–22^.

In the present paper, we first optimized the triplex DNA pull-down procedure with naked plasmids by introducing a relatively extended linker between the TFO itself and a desthiobiotin residue as to efficiently form a complex with streptavidin-coated matrices. The procedure was then applied to various experimental set-ups *in vitro* and *in vivo*. The approach is shown to allow pull-down of proteins associated with plasmids hosted in living *E. coli* cells. It was also successfully applied to the recovery of a nucleosome reconstituted *in vitro* opening many possibilities to study post translational modifications of histones in the context of a genuine nucleosome. Incubation in human nuclear extracts of a plasmid carrying a transcriptionally active NF-κB model promoter is shown to capture specific transcription factors. Finally, we successfully applied this approach to isolate a specific chromatin fragment from human cells. The data reveals that the approach allows the isolation a specific chromatin fragment from the large bulk of undesired genomic DNA with a signal over noise ratio ≥ 10^5^. In conclusion, the triplex mediated DNA capture assay developed here is particularly versatile as it can be implemented with any DNA sequence-of-interest or native plasmids, (i.e., with no prior modification), that contain a short sequence tag for triplex formation with a cognate oligonucleotide probe. The approach can also be applied to any DNA adduct or secondary structure (hairpins, quadruplexes, triplet repeats and so on).

## Results and Discussion

The establishment of triplex formation between an oligonucleotide and dsDNA involves hydrogen bond formation (Hoogsteen base-pairing) within the major groove of the helix. This unique feature allows sequence-specific recognition of DNA without the need of denaturing the Watson-Crick base pairs as in traditional hybridization protocols. Our goal is to develop a robust experimental workflow that makes use of the unique feature of triplex formation to capture, identify and characterize proteins assembled, *in vivo* or *in vitro*, on a given DNA sequence-of-interest. The basic approach is to capture sequence-specific protein/DNA complexes, followed by affinity purification on streptavidin-conjugated matrices and by analysis of the purified nucleoprotein complexes (i.e., DNA and proteins).

### TFO-mediated Plasmid Capture (TmPC) *in vitro*

We initially attempted to prepare biotin-conjugated plasmids through direct biotinylation; various trials turned out to be unsatisfactory (see Supplementary information and Supplementary Fig. S1) most probably as a result of extensive steric hindrance between plasmid and beads that strongly limits capture efficiency. Moreover, to make our approach adaptable to many applications, we aimed at a technique able to capture native plasmids (i.e., with no prior modification). For this purpose we choose to develop a capture tool based on the formation of a triplex between an oligonucleotide probe (i.e., TFO) and a specific sequence tag (Triplex Forming Tag: TFT) present within the DNA molecules to be pulled-down.

**Figure 1:**
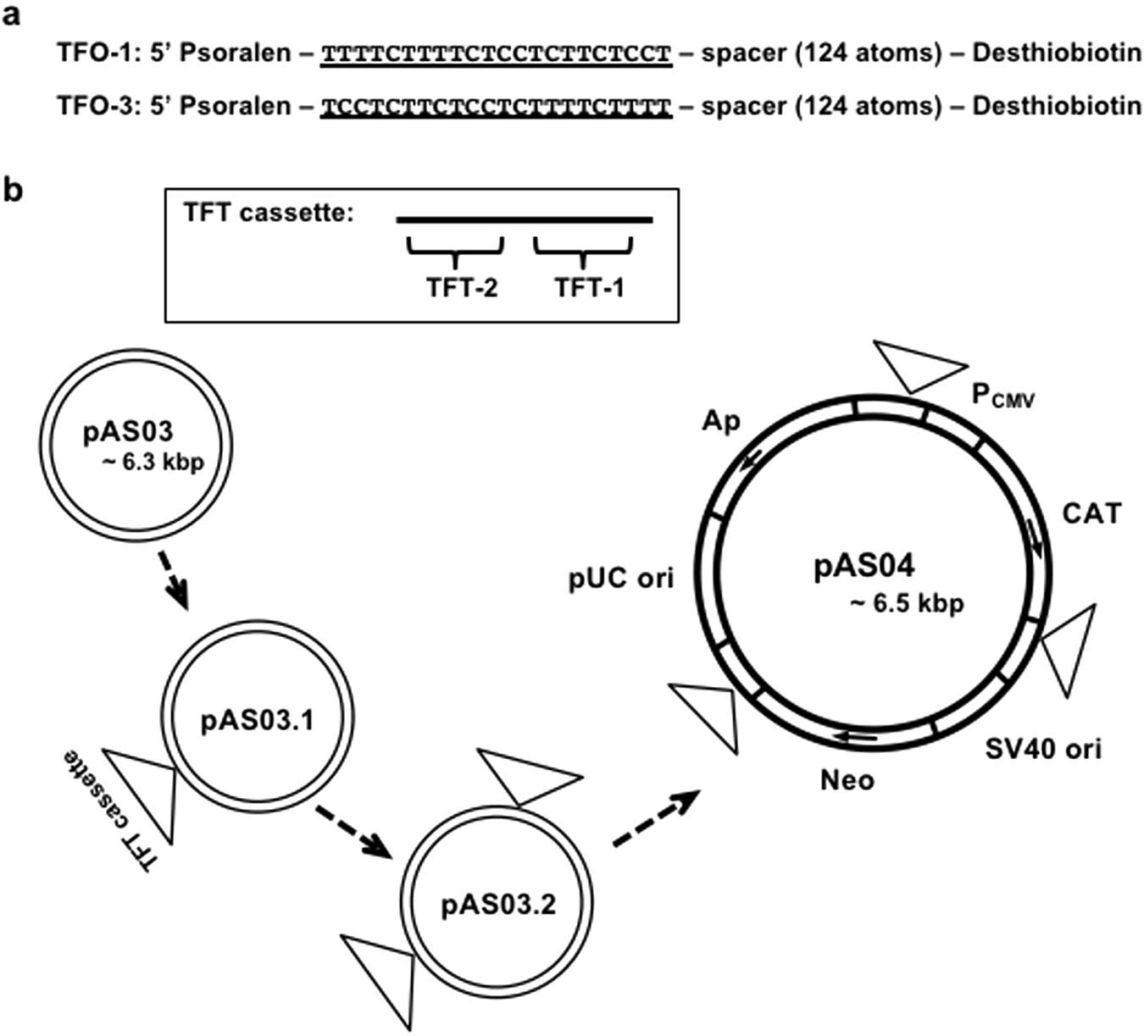
Structure of the TFO probes and the TFT cassettes containing constructs: (**a**) TFO probe structure: the specificity head (underlined) is formed by a 22-mer oligonucleotide composed of a mixture of LNA and DNA residues. A psoralen residue is grafted at the 5′-end of the oligonucleotide. Its 3′-end is modified with a desthiobiotin residue attached to spacer composed of a linear chain of 124 atoms. (**b**) Construction of plasmids containing a Triplex Forming Tag (TFT) cassette: pAS03 is derived from pcDNA3.1(+)CAT. pAS03.1 is derived from pAS03 by inserting a TFT cassette containing two different TFO-target sites (TFT-1 and TFT-2). pAS03.2 is derived from pAS03.1 by inserting an additional TFT cassette. pAS04 is derived from pAS03.2 by inserting a third TFT cassette. pUC ori: high copy number origin in *E. coli*. SV40 ori: replication origin in primate cells expressing SV40 large T antigen. Ap: ampicillin resistance gene. Neo: neomycin resistance gene. CAT: chloramphenicol resistance gene. P_CMV_: human cytomegalovirus immediate-early promoter/enhancer.

First, we wanted to establish a robust procedure to capture naked plasmid DNA *in vitro*, as this step appears to be central to all further applications. We designed a molecular probe, TFO-1 (Fig. 1a), constituted of the following elements: 1) a 22-mer oligonucleotide composed of a LNA/DNA mixture; its sequence is complementary to the TFT cassette present in the dsDNA to form a triplex; 2) a psoralen residue attached at the 5′-end of the oligonucleotide contributes to triplex stabilization; 3) a desthiobiotin residue attached at the 3′-end via a long spacer; this residue can interact with streptavidin; the weaker desthiobiotin-streptavidin interaction allows competitive biotin elution of the captured plasmid. We also designed TFO-3 whose components are the same as TFO-1 except for the sequence of the 22-mer oligonucleotide (Fig. 1a). A short TFT cassette is introduced into the DNA molecules to be captured. The cassette contains two TFTs, TFT-1 and TFT-2 that are designed to form triplexes with TFO-1 and TFO-3, respectively. The resulting plasmids (pAS03 ~ pAS04) (Fig. 1b) are amplified in *Escherichia coli* (*E. coli*), purified and used in TFO-mediated Plasmid Capture (TmPC) experiments as substrates.

**Figure 2:**
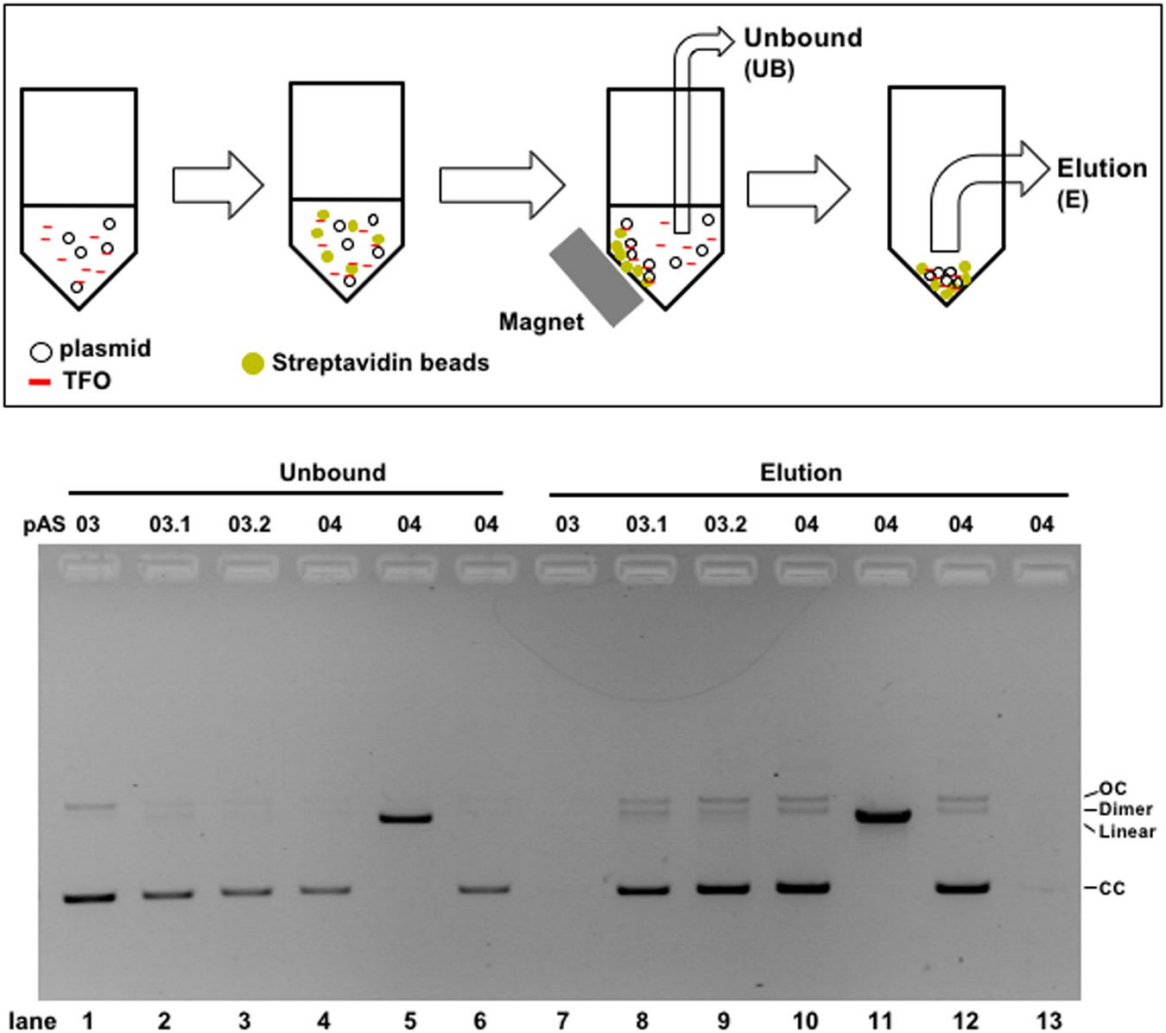
Plasmid capture *in vitro* via TFO-mediated triplex formation: 600 ng (≈140 fmol) of each plasmid (pAS03, pAS03.1, pAS03.2, pAS04) are mixed with 8 pmol of TFO-1 for 24 hr, followed by mixing with streptavidin magnetic beads. The plasmid fraction not bound to beads (unbound: UB) is recovered using a magnetic stand (lanes 1–6). After the washing steps, plasmid bound to the beads is released either by boiling (lanes 7–11) or by competitive elution using a biotin-containing buffer (lane 12). Following competitive elution, the beads are re-suspended in a buffer and further eluted by boiling (lane 13). All plasmids are supercoiled circular except for lanes 5 and 11 where the plasmid is linearized. The recovered samples are analysed by agarose gel electrophoresis. Similar results were obtained in three independent experiments and a representative gel picture is shown here. In addition, TFO-3 was shown to perform similarly (data not shown). The loading amounts are 15 ng (lanes 1–6), 30 ng (lanes 7–12), and 60 ng (lane 13) plasmid equivalent. Estimation of plasmid recovery compared to input is: pAS03, <1% (lane 7); pAS03.1, ≈50% (lane 8); pAS03.2, ≈60% (lane 9); pAS04 (lanes 10–12), ≈70%, as quantified by NIH ImageJ software. CC: closed circular. OC: open circular. Dimer: dimerized plasmid.

In order to test the functionality of our probe for capturing plasmids, the TFO-1 probe is incubated with a given plasmid containing or not the TFT sequence for triplex formation and then mixed with streptavidin-coated magnetic beads. Beads are collected via a magnet and the supernatants (UB: unbound fraction) are collected for analysis. After washing of the beads, the plasmid fraction bound to beads is eluted by heat treatment and analysed by agarose gel electrophoresis (ethidium bromide (EtBr) stained gel) together with the UB fraction. As shown in Fig. 2, the presence of plasmids in the eluted fraction (E) depends upon the presence of a TFT cassette. Its recovery appears to be independent of the DNA structure as ccDNA and linear DNA are recovered equally (lanes 10 vs 11). The recovery yield increases when the number of TFT cassette in the plasmid increases from 1 to 3 to reach a limit of about 70% of input DNA. This limit is likely to reflect the presence of un-perfectly synthesized TFO probe. Increasing the number of TFT sites per plasmid partially compensates for the negative effect caused by TFO impurities. As expected, there is a reciprocal relationship in the amount of plasmid detected in UB and E fractions (lanes 1–6 vs 7–12). Elution with excess free biotin leads to a similar amount of plasmid recovery (lane 12) in good agreement with the fact that further treatment of the biotin-eluted beads with heat does not yield additional DNA (lane 13).

### Isolation of DNA Associated Proteins (IDAP)

Comprehensive analysis of proteins bound directly or indirectly to specific DNA sequences represents a fundamentally important goal. As outlined below, feasibility of the triplex-mediated approach for capture of nucleoprotein complexes has been evaluated *in vivo* and *in vitro* in the following examples: 1. in living *E. coli*; 2. in a reconstituted mono-nucleosome; 3. in human nuclear extracts.

### Living *E. coli*

After optimizing the capture of naked plasmids *in vitro*, we wanted to test the approach using a physiologically relevant biological sample. To this end we chose *E. coli*’s strains transformed with plasmid pAS04 (with TFT) or pAS03 (without TFT). Strains DH1/pAS03 and DH1/pAS04 are cultivated to either log or stationary phase and cross-linked with formaldehyde. Cross-linked cells are disrupted, followed by centrifugation for removal of insoluble materials. The resulting supernatant is used as an input sample. Both TFO-1 and TFO-3 are incubated with the input sample and further processed as in the above-described TmPC protocol except for the use of different washing conditions (Supplementary Fig. S2a). After reverse crosslinking, analysis of the eluted proteins by PAGE (polyacrylamide gel electrophoresis) separation followed by silver-staining reveals a protein profile that is significantly enriched in amount and complexity in the pAS04-derived fraction compared to pAS03 illustrating the specificity of the capture protocol (Supplementary Fig. S2b). In good agreement with the protein profiles, only plasmid pAS04 is detected in both log and stationary phases illustrating the high specificity of the capture protocol (Supplementary Fig. S2c). Overall these data suggest that the majority of proteins isolated with pAS04 are indeed bound to plasmid pAS04. Our IDAP approach is thus likely compatible with material derived from living cells.

### Reconstituted mono-nucleosomes

A typical *in vitro* approach for analysing specific protein/DNA complexes is to use as a substrate an oligonucleotide that carries a Sequence-of-Interest (e.g., as in gel-shift assay). We aimed to assess whether specific nucleoprotein complexes assembled *in vitro* could be isolated and analysed using the triplex-mediated methodology (Supplementary Fig. S3a). We choose to establish the feasibility of the approach with a reconstituted mono-nucleosome^23,24^. We designed a synthetic dsDNA, RN01, as the substrate for nucleosome reconstitution (Supplementary Fig. S3b). The central region of RN01 consists of a nucleosome positioning sequence (NPS) that favours precise nucleosome localization^25–28^. Both ends of RN01 contain TFT-1 and TFT-2 tags to target triplex formation with TFO-1 and TFO-3, respectively. The length of RN01 (209 bp) exclusively supports formation of a mono-nucleosome particle as 147 bp DNA fragment is wrapped around the histone octamer. As the histone core bound to a NPS sequence is known to be relatively stable^26,27^, it is expected that the TFT sites at both ends of RN01 will be accessible for efficient triplex formation^29^. We first verified that naked RN01 is efficiently captured by the TFO probes (≈25% recovery) (Supplementary Fig. S3c).

**Figure 3:**
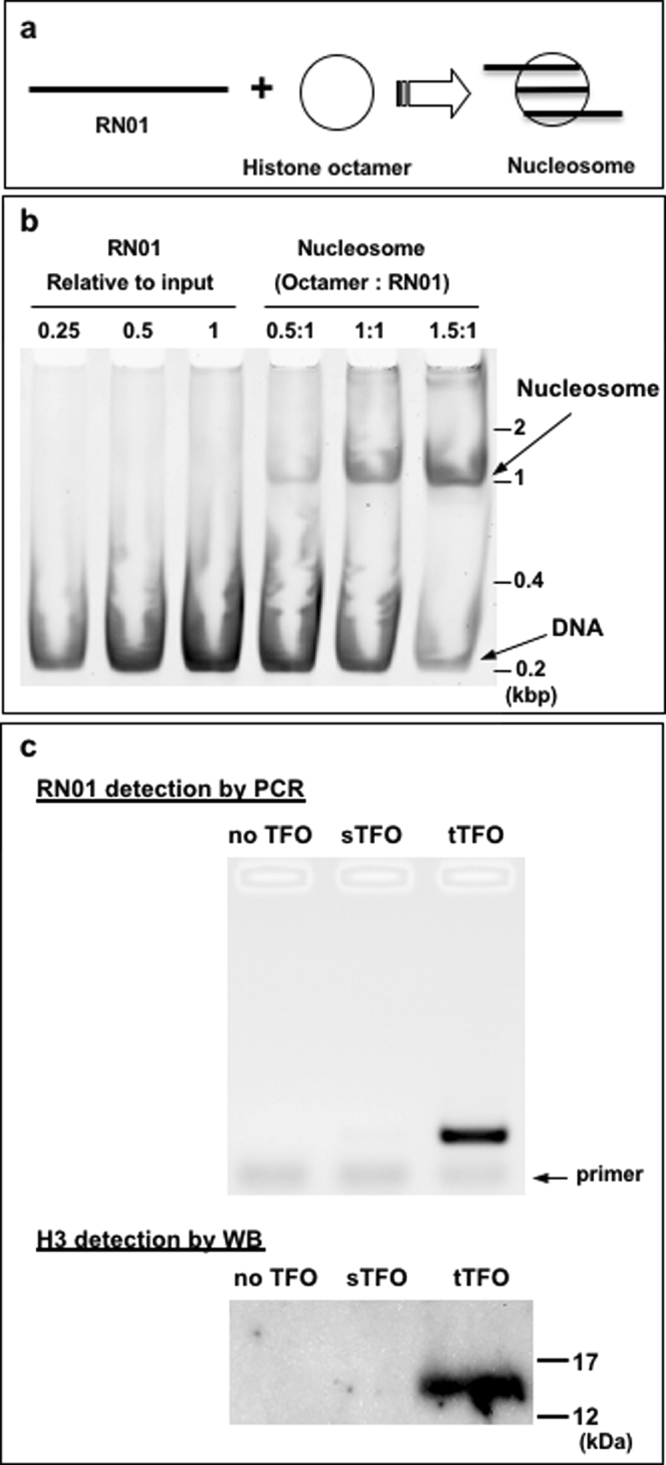
Nucleosome capture depends upon the presence of tTFO: (**a**) Schematic view of reconstitution of mono-nucleosome. (**b**) A double-stranded (209 bp) DNA (RN01) is used for reconstituting mono-nucleosomes *in vitro*. RN01 is incubated with the component of the histone core (i.e., human histones H2A, H2B, H3, and H4). Gel shift assay is carried out to assess mono-nucleosome reconstitution: octamer / DNA ratio ranging from 0.5 to 1.5. The results show that >50% of DNA is incorporated into mono nucleosomes at octamer/DNA ratio equal to one or above. (**c**) Cross-linked RN01 nucleosomes are incubated with buffer (no TFO), scrambled TFO (sTFO: a mixture of Scr-1 and Scr-2) the sequences unable to form a triplex with the TFT cassette, or target TFO (tTFO: a mixture of TFO-1 and TFO-3) complementary to the TFT cassette for triplex formation. These samples are subsequently incubated with streptavidin-conjugated beads. Beads are washed and bound material is eluted, followed by heat to reverse the cross-links. A small fraction (0.001%) of each elution product is amplified by PCR for DNA detection, followed by quantification using Image Lab software (Bio-Rad). PCR quantification was performed as detailed in Fig. 6d. We performed three independent experiments that all led to results similar to those shown in the present figure. For western blotting (WB), 80% of the elution products are used for histone H3 detection. Full-length blots are presented in Supplementary Fig. S8.

Human histones are incubated with RN01 to reconstitute a mono-nucleosome (Fig. 3a). A major fraction of RN01 is converted into a mono-nucleosome assembly when the ratio histone octamer over RN01 is either stoichiometric or in slight excess (Fig. 3b). The reconstituted nucleosome is first cross-linked with formaldehyde as nucleosomes become unstable upon dilution^30,31^. Cross-linked nucleosomes are incubated with target TFO (tTFO: can form triplex), with scrambled TFO (sTFO: cannot form triplex), or with buffer (no TFO), followed by incubation with streptavidin-conjugated beads. Following washes of the beads, the material bound to the beads is competitively eluted via a biotin-containing buffer. RN01 DNA is assayed in the eluted fraction by PCR (Fig. 3c), a positive signal is only seen when tTFO was used during capture (≈30% recovery). Similarly, a clear signal is only observed in lane tTFO by western blotting (WB) analysis using an anti-histone H3 antibody (Fig. 3c).

These data illustrate the feasibility of our approach in the context of a reconstituted nucleosome (i.e., a relatively complex nucleoprotein particle). It opens the possibility to study specific histone modifications by purified epigenetic enzymes in the context of a genuine chromatin component. This approach is compatible with high-throughput assays in the context of drug screening provided fluorescent labelling is implemented.

### Human nuclear extracts

**Figure 4:**
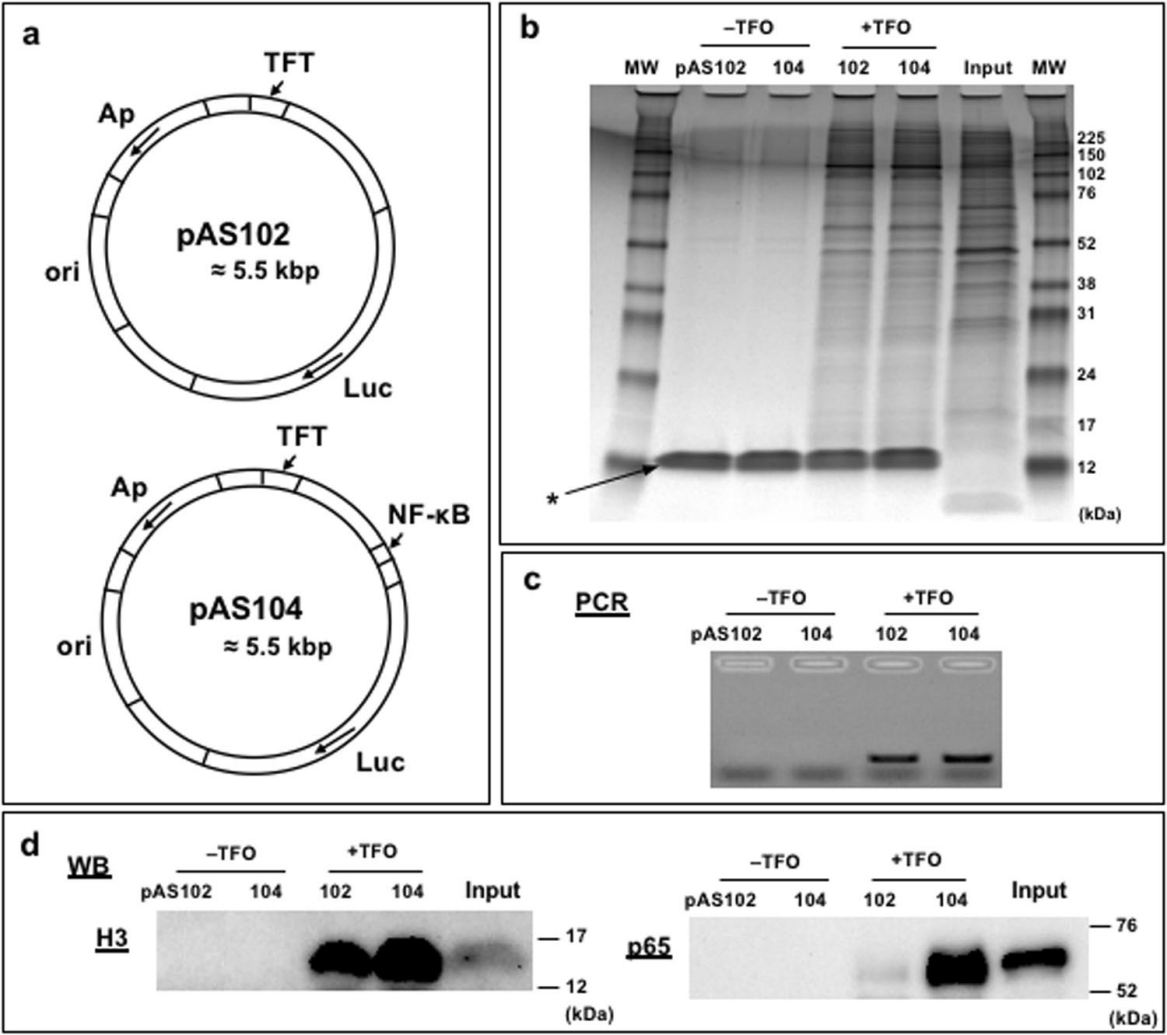
Isolation of NF-κB proteins from human nuclear extracts via IDAP: (**a**) Schematic structure of the plasmids. A model NF-κB binding site is only present in plasmid pAS104, not in pAS102. ori: high copy number pUC origin in *E. coli*. Luc: Luciferase gene for a reporter assay. Ap: ampicillin resistance gene. TFT: Triplex Forming Tag. NF-κB: a binding site for NF-κB proteins. (**b**) Protein detection via silver staining. 80 ng of plasmids are incubated with ≈ 2.4 × 10^6^ cells equivalent of human nuclear extracts. 20% of the elution products are loaded. Lanes are pAS102 or pAS104 in absence or presence of TFO. Input (nuclear extracts) from ≈ 2.5 × 10^4^ cells is loaded. MW: molecular weight marker. *: streptavidin (**c**) Plasmid detection via PCR. PCR quantification was performed as detailed in Fig. 6d. 0.003% of the elution products are used as a template. (**d**) Protein detection via WB. For histone H3 detection: 50% of the elution products and Input (nuclear extracts) from ≈ 1 × 10^5^ cells are loaded; For p65 detection: 22% of the elution products and Input (nuclear extracts) from ≈ 2.5 × 10^4^ cells are loaded. The set of experiments (b ~ d) were independently performed four times with similar results. Full-length blots are presented in Supplementary Fig. S8.

Next, we chose to apply the protein-DNA capture approach to human nuclear extracts. As a Proof-of-Concept, we chose to investigate the possibility to isolate transcription factors belonging to the NF-κB pathway that is extensively studied in view of its importance in many human health issues^32,33^. As the pathway is activated by various stimuli such as TNFα, we chose to prepare human nuclear extracts from TNFα-stimulated cells. We constructed plasmid pAS104 that contains the TFT cassette located near a promoter containing the NF-κB consensus binding site (Fig. 4a). Plasmid pAS104 is first mixed with TFO-1 probe to form the triplex followed by UVA irradiation to introduce a covalent crosslink between the plasmid and the psoralen moiety of TFO-1. The pAS104-conjugated TFO or pAS104 (in the absence of added TFO) is incubated with streptavidin beads. After washing of the beads to remove unbound plasmid, the pAS104-immobilized plasmid is mixed with the human nuclear extracts. Following washes of the beads, protein/DNA complexes are eluted from the beads by heat. As a negative NF-κB control, we performed the same assay with plasmid pAS102 that is identical to pAS104 but lacks the NF-κB binding site (Fig. 4a).

When analysing the eluted proteins by silver staining via PAGE, a large number of proteins are detected in a TFO-dependent way (Fig. 4b). Our washing conditions appear to efficiently remove non-specific binding proteins to beads as shown in the −TFO control (Fig. 4b). Proteins in the +TFO lanes essentially thus represent plasmid-bound proteins. This point is additionally proven by PCR analysis that reveals the presence of plasmid sequences only in the +TFO lanes (Fig. 4c). The presence of histone H3 is also specifically revealed in the +TFO lanes for both pAS102 and pAS104 (Fig. 4d), illustrating robust capture specificity. Interestingly, protein p65 (RelA), the hallmark in the NF-κB proteins, is significantly enriched in pAS104 compared to pAS102 (Fig. 4d). This result shows that NF-κB proteins bound to the cognate binding site in pAS104 have been specifically captured.

In general, various enhancers regulate promoters during transcription^34^. Our assay system (IDAP) could be fully compatible to analyse relatively long DNA targets (e.g., an enhancer being located at several kbp apart from a promoter). Taken together, the IDAP methodology is likely to be adaptable to all types of DNA-protein interactions that occur during transcription, repair, replication and recombination.

### Isolation of specific chromatin fragments from cells: Chromatin-of-Interest Fragment Isolation (CoIFI)

**Figure 5:**
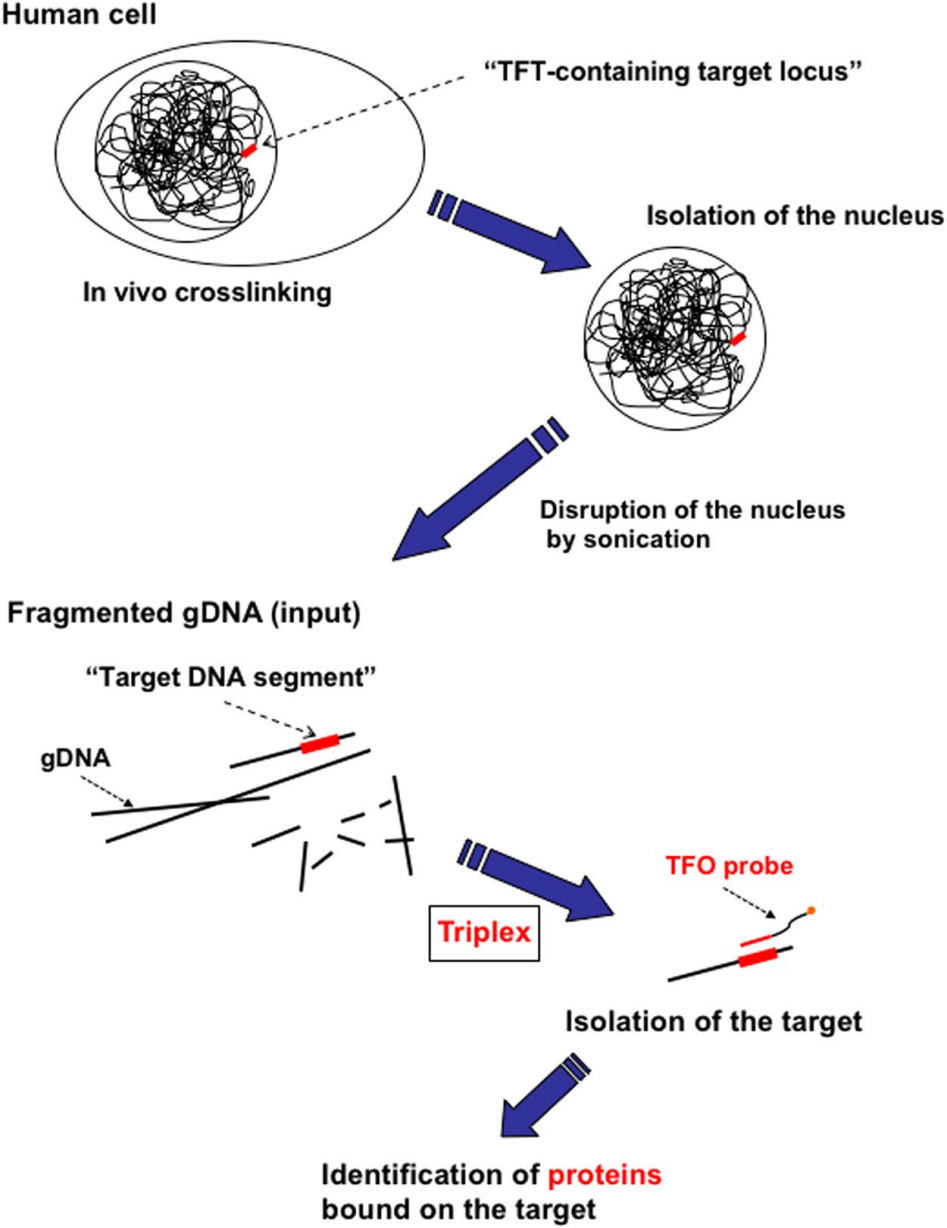
Schematic outline of the CoIFI procedure.

**Figure 6:**
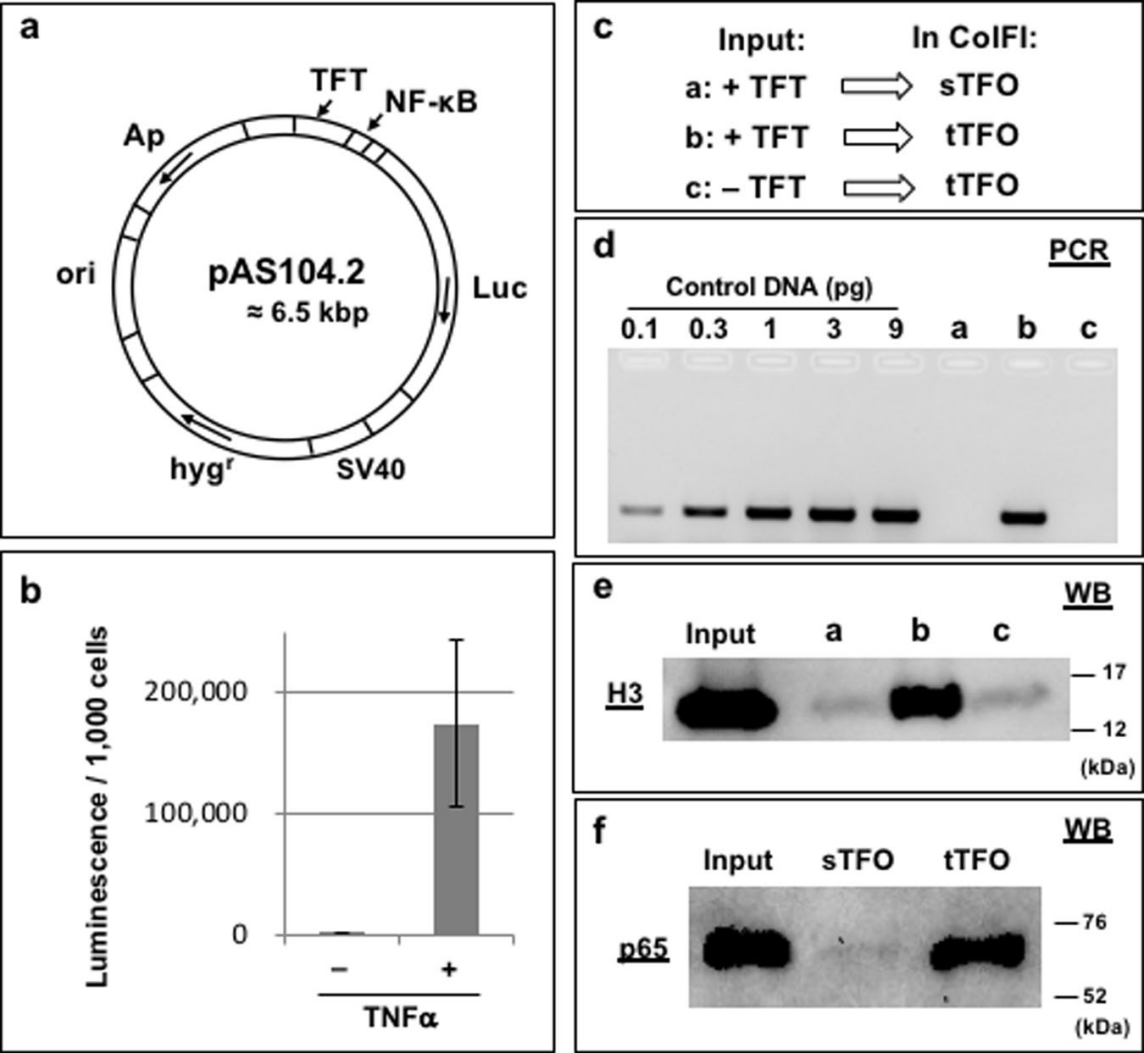
Feasibility of the CoIFI approach using a stably transformed cell line: (**a**) Schematic structure of the plasmid used to obtain stable human cell lines. ori: high copy number pUC origin in *E. coli*. Luc: Luciferase gene for a reporter assay. Ap: ampicillin resistance gene. TFT: Triplex Forming Tag. SV40: SV40 early enhancer/promoter. hyg^r^: hygromycin resistance gene. NF-κB: a binding site for NF-κB proteins. (**b**) Reporter assay based on Luciferase activity. The G69 cells are stimulated by exposure to 10 ng/ml of TNFα for 2 hr; luminescence is measured by a luminometer. The data, derived from three independent assays, are expressed as relative light unit for non-stimulated cells (−TNFα) and stimulated cells (+TNFα). (**c**) Experimental design: Inputs are prepared from ≈0.9 × 10^6^ cells equivalent of cell lines containing the TFT site (+TFT; clone G69) or not (−TFT; clone c2-1). Input samples are incubated with either sTFO (scrambled sequence) or tTFO (target sequence). Together, three experimental conditions designed as a, b, c are tested (see main text for additional explanations). (**d**) Plasmid detection via PCR. 2% of the elution products are used as a template to amplify a DNA segment in the luciferase gene. Lanes **a**–**c** correspond to the conditions outlined in Fig. 6c. The indicated amounts of control plasmid pAS104.2, were similarly amplified. Quantification of the bands by Image Lab software (Bio-Rad) produces the standard curve shown in Supplementary Fig. S7. This experiment was performed independently five times and led to similar results. (**e**) Histone H3 detection via WB. ≈83% of the elution products and input from ≈ 50 cells equivalent are loaded. Lanes a~c correspond to the conditions outlined in Fig. 6c. (**f**) p65 detection via WB. Inputs are prepared from ≈2.5 × 10^7^ cells equivalent of clone G69 cell line. Extracts are incubated with either sTFO or tTFO. Input lane is loaded ≈ 200 cells equivalent. Full-length blots are presented in Supplementary Fig. S8.

One of our major goals is to isolate protein/DNA complexes assembled on a sequence-of-interest within the context of chromatin in the human genome. Compared to the capture of proteins in the context of human nuclear extracts that specifically binds to a DNA sequence in a plasmid (i.e., the IDAP procedure described above), the capture of a specific chromatin fragment is challenged by the huge excess of non-target chromatin fragments. As a proof-of-concept, in order to increase the signal over noise ratio, we constructed cell lines stably transformed with multiple copies (see below) of a construct that carries a sequence-of-interest linked to a TFT cassette. The overall strategy of the approach is referred to as Chromatin-of-Interest Fragment Isolation (CoIFI) (Fig. 5). We constructed plasmid pAS104.2 carrying a luciferase gene fused to a promoter containing the NF-κB binding site and a hygromycin resistance cassette for selection (Fig. 6a). Stably transformed human cell lines were established by transfection 293 cells by plasmid pAS104.2 and selection on hygromycin. A stable cell line, called clone G69, that contains ≈160 copies of the construct integrated into the genome was chosen (Supplementary Fig. S4). The NF-κB promoter region was shown to be transcriptionally functional as luciferase mediated fluorescence was found to be highly induced by TNFα treatment (Fig. 6b). We prepared an input sample from clone G69 cells for which the amount of integrated DNA construct represents about 0.0067% of total genomic DNA (gDNA) (Supplementary Fig. S5). The pattern of gDNA in the input exhibits a broad distribution of fragmented gDNA (Supplementary Fig. S6a). If the average size of the fragmented gDNA is ≈2 kbp, DNA fragment from the NF-κB construct with the TFT site will represent ≈0.002% of total DNA (0.0067% x 2/6.5 = ≈0.002%). Therefore, a ≥ 10^5^-fold enrichment of construct-derived DNA over bulk gDNA fragments will be required during fragment capture experiment in order to be able to specifically analyse proteins bound to the sequence-of-interest (Supplementary Fig. S6b). To evaluate the specificity of the CoIFI protocol, we implemented the following three experimental conditions (outlined in Supplementary Fig. S6c): we prepared an input sample from cell line G69 containing the NF-κB construct linked to a TFT cassette. This input sample was subjected to the CoIFI protocol using either the target TFO probe (condition b) or, as a negative control, the scrambled version of the sequence (condition a) (Fig. 6c). We set up an additional negative control using an input sample from a stable cell line with multiple copies of the construct lacking the TFT cassette (clone c2-1; ≈ 350 copies) challenged with the target TFO probe (condition c) (Fig. 6c). The material captured under all three conditions was subjected to DNA analysis (PCR amplification of a sequence present in the integrated construct) and to protein detection (WB analysis). For DNA analysis, only sample from condition b, exhibits a specific PCR amplification signal (lane b in Fig. 6d). We estimated the amount of construct DNA that we recovered to be in the range of 15% of the theoretical amount present in the input sample (≈15% recovery = ≈23 amol equivalent of target DNA segments). For protein analysis, we thought to use histone detection, as histones are undoubtedly among the most common chromatin proteins. Histone H3 present in as little as 50 cells is clearly detected by WB (lane Input in Fig. 6e). As outlined in Supplementary Fig. S6c, if the enrichment factor is less than 10^5^-fold we expect to see a WB signal for H3 in all three conditions. In contrast, if the signal/noise (s/n) ratio is more than 10^5^-fold, the H3 specific band should preferentially be detected under condition b only. As shown in Fig. 6e, histone H3 is well detected under condition b only, i.e. when the correct combination of input sample and TFO was used, indicating a remarkable ≥ 10^5^-fold s/n ratio. We thus anticipate that the majority of proteins captured by the CoIFI protocol are bound to the stably integrated construct (i.e., sequence-of-interest). Indeed, analysis by WB specifically shows the presence of p65 (RelA) when cells from clone G69 are subjected to CoIFI mediated capture are analysed. The p65 band is not observed when the scrambled sTFO probe was used in the capture experiment attesting the robust specificity of the present methodology.

It should be noted that the capture of dsDNA by an oligonucleotide via Watson-Crick base-pair formation as in PICh (Proteomics of Isolated Chromatin segments) protocol as pioneered by the Kingston team requires several partial heat denaturation steps that alter chromatin architecture and composition^7^. Later, the same team clearly demonstrated that target sites to form Watson-Crick base-pairs with oligonucleotides absolutely need to be located at ends of dsDNA since partial heat denaturation is only effective at ends^11^. In contrast, the triplex-mediated capture methodology described here proceeds via Hoogsteen base pair formation in the major DNA groove without any heat denaturation step and is not restricted to DNA ends. We also note that the signal over noise ratio (s/n) reached in classical immunoprecipitation-based protocols like ChIP is in the range of ~10^2^, while PICh (or ePICh) protocols^11^ achieve a much better s/n ratio of ≈10^4^. As shown here, the s/n ratio in the CoIFI procedure is further improved to ≥10^5^.

## Conclusions

The newly developed procedure (i.e., IDAP and the related protocols) can easily be adapted to diverse experimental situations, using either native plasmids or short double-stranded DNA fragments. For instance, a plasmid containing a sequence-of-interest (e.g., a promoter) can first be mixed with the TFO probe to form the triplex (that can be converted into a covalently bond form through psoralen photo cross-link reaction). The preformed TFO-plasmid conjugate can be incubated in crude cellular extracts and the proteins bound to any sequence-of-interest, DNA adduct or DNA secondary structure can subsequently be pulled down and analysed by proteomics. Alternatively, plasmids, viral replication or transcription complexes present in cells can be captured via triplex formation and analysed by proteomics. The approach is compatible for studying biochemical reactions in reconstituted nucleoprotein complexes such as nucleosomes. Certain histone methyltransferases exhibit different activities depending on the presentation of the substrate as a free histone or within the nucleosome structure^35^. Screening of chemical libraries for the search of histone methyltransferases (EZH1, EZH2) inhibitors, was successfully conducted using reconstituted nucleosomes as substrates^36^. Our approach allows histone modifications by epigenetic enzymes to be studied on genuine nucleosome structure. When combined with time-resolved fluorescence approaches it would allow high throughput screening of small molecules able to modulate specific chromatin-modifying proteins^37,38^. In addition, the present approach allows side-by-side comparisons of the proteins assembled to a sequence-of-interest in the context of nuclear extracts or in a cell nucleus. Moreover, if a plasmid containing a sequence-of-interest (e.g., promoter, enhancer, etc.) linked to a TFT cassette is introduced into a stem cell (e.g., human induced pluripotent stem cell (iPSC), human embryonic stem cell (ESC)), it will be possible to monitor protein complexes assembled in various cell types derived from differentiation of a unique parental cell. In addition, the approach allows us to monitor the status of the sequence-of-interest itself by next generation sequencing (NGS) techniques (e.g., epigenetic DNA modification, genome stability).

## Methods

### Capture tools and DNA

All TFO probes are composed of a psoralen residue, a 22-mer LNA/DNA mixed oligonucleotide, a spacer arm composed of tandemly oriented hexaethylene glycol, and a desthiobiotin residue as represented in Fig. 1a. Sequence contexts of the 22-mer oligonucleotides are as follows: TFO-1, 5′-tTtTcTtTtCtCCtCtTCtCct; TOF-3, 5′-tCcTcTtCtCcTcTtTtCtTtt; Scr-1, 5′-tCtCtCtTcTtCtTcCtTcTcc; Scr-2, 5′-tTcTtCtTcCtCtTtCcTcCtc. LNA and DNA residues are shown in small and capital letters, respectively. Capital C represents 5-methyl dC residue. A TFT cassette is 61 bp dsDNA and contains two different TFO-target sites (TFT-1 and TFT-2). TFT-1 is 5′-AAAAGAAAAGAGGAGAAGAGGA and TFT-2 is 5′- AGGAGAAGAGGAGAAAAGAAAA. pAS03 is derived from pcDNA3.1(+)CAT (Invitrogen). pAS03.1 is derived from pAS03 by inserting a TFT cassette at a BstBI site. pAS03.2 is derived from pAS03.1 by inserting an additional TFT cassette at a BglII site. pAS04 is derived from pAS03.2 by inserting a third TFT cassette at an XbaI site. Linearized pAS04 is prepared by a unique cut using EcoRV at a site located between CMV promoter and CAT gene. RN01 is prepared by PCR of an NPS region (referred to as 601) on pGEM-3z/601 (Addgene)^26^. Primers for the PCR contain TFT-1 and TFT-2 in each primer. The RN01 sequence context is as follows: 5′- GCTTTAAAAGAAAAGAGGAGAAGAGGAATGCACAGGATGTATATATCTGACACGTGCCTGGAGACTAGGGAGTAATCCCCTTGGCGGTTAAAACGCGGGGGACAGCGCGTACGTGCGTTTAAGCGGTGCTAGAGCTGTCTACGACCAATTGAGCGGCCTCGGCACCGGGATTCTCCAGGGAATAGGAGAAGAGGAGAAAAGAAAATACG. All oligonucleotides are purchased from Eurogentec. pAS102 is derived from pGL4.12 (Promega). This plasmid contains TFT inserted upstream of Luciferase gene; the TFT is composed of 6 tandemly repeated TFT cassettes. pAS104 is derived from pAS102 by inserting a NF-κB binding site between the TFT and the Luciferase gene. The NF-κB binding site (5′-GGGAATTTCC) is derived from a region between NheI and BsrGI of pGL4.32 (Promega). pAS104.2 is derived from pAS104. This plasmid contains a hygromycin resistant gene derived from a region between BamHI and SalI of pGL4.32 (Promega). Random biotinylation and quantification of the amount of bound biotin to plasmid pAS03 is carried out following the manufacturer’s instructions (VECTOR laboratories, Burlingame, CA). Capture of randomly biotinylated DNA is carried out following the manufacture’s instruction in Dynabeads kilobaseBINDER kit (Invitrogen).

### TFO-mediated plasmid capture (TmPC)

Plasmid is mixed with TFO-1 for 24 hr in buffer RW (25 mM Tris-Cl (7.6), 0.1 mM EDTA, 0.5 mM EGTA, 150 mM NaCl, 1% NP40, 1% Na deoxycholate, 0.1% Sarkosyl, 0.1% SDS) with 10 mM MgCl_2_. Dynabeads MyOne Streptavidin C1 (Invitrogen) are added to the mixture and agitated for 30 min at 25 °C. The mixture is placed on a magnetic stand and the C1-bound fraction is collected. The collected C1 beads are washed twice with buffer RW and plasmid captured on the C1 is released by either boiling in buffer RW or competitive elution using buffer E (12.5 mM Tris-Cl (7.6), 0.1 mM EDTA, 0.5 mM EGTA, 75 mM NaCl, 0.5% NP40, 0.5% Na deoxycholate, 0.1% Sarkosyl, 0.05% SDS, 10 mM D-biotin) for 1 hr. Quantification of plasmid recovery is deduced by comparison with known amounts of control DNA loaded on gel.

### IDAP in *E. coli*

See Supplementary information

### TFO-mediated RN01 capture

See Supplementary information

### Reconstitution of mono-nucleosome

See Supplementary information

### IDAP in mono-nucleosome

See Supplementary information

### IDAP in human nuclear extracts

Human nuclear extracts are prepared from 293 F (Invitrogen). The procedure is derived from the manufacture’s instruction of CelLytic NUCLEAR Extraction kit (Sigma) and^39^. The 293 F cells are stimulated with 10 ng/ml TNFα for 2 hr in a flask. The stimulated 293 F cells (2~4 × 10^7^ cells) are re-suspended with 5 packed cell volume of CM-Lysis buffer (10 mM HEPES (7.9), 10 mM KCl, 1.5 mM MgCl_2_, 1 mM DTT, 0.3 M Sucrose, 1% NP40, 0.1 mM EDTA, Protease inhibitor cocktail (Sigma)) via pipetting. This is overlaid on 1 ml of SG buffer (10 mM HEPES (7.9), 10 mM KCl, 1.5 mM MgCl_2_, 1 mM DTT, 1.5 M Sucrose, 0.1 mM EDTA, Protease inhibitor cocktail), followed by centrifugation. The nuclear pellet is re-suspended with NW buffer (10 mM HEPES (7.9), 10 mM KCl, 1.5 mM MgCl_2_, 1 mM DTT, 0.1 mM EDTA, Protease inhibitor cocktail), followed by centrifugation. The pellet is re-suspended with 24 μl of Extraction buffer (20 mM HEPES (7.9), 1.5 mM MgCl_2_, 0.42 M NaCl, 1 mM DTT, 0.2 mM EDTA, 25% (v/v) Glycerol, Protease inhibitor cocktail), and incubated on ice for 1 hr. The nuclear extracts are recovered by centrifugation and exposed to streptavidin magnetic beads in order to remove proteins that bind to beads. The resultant supernatant is used as nuclear extracts in assays. For preparation of plasmid with TFO, 600 ng of pAS102 or pAS104 are mixed with 3 pmol of TFO-1 for 18 hr in buffer TF2 (10 mM Tris-Cl (7.5), 70 mM NaCl, 0.02% NP40) with 10 mM MgCl_2_ at 25 °C. The mixture is irradiated for 0.2 J/cm^2^ at 365 nm light to introduce crosslink between the psoralen in TFO probe and plasmid. This plasmid with TFO (or plasmid without TFO as a control) is mixed with Dynabeads M-280 Streptavidin (Invitrogen) for 30 min in buffer NCL (20 mM Tris-Cl (7.5), 4% glycerol, 8 mM DTT, 4 mM MgCl_2_, 0.5 mM EDTA, 0.1% NP40) with 250 mM NaCl and 1.25 mg/ml BSA. The beads are washed three times with buffer NCL with 250 mM NaCl, and two times with buffer NCL with 50 mM NaCl. Nuclear extracts are added to the beads and incubated for 1 hr in buffer PM2 (20 mM Tris-Cl (7.5), 4% glycerol, 8 mM DTT, 4 mM MgCl_2_, 2.5 mM ATP, 50 mM NaCl, 0.02% NP40) at 25 °C. The mixture is placed on a magnetic stand and the fraction bound to beads is collected. The collected beads are washed five times with buffer NCL with 75 mM NaCl. Protein/DNA complexes captured on the beads are released by boiling in buffer CB3. For western blotting, an anti-histone H3 antibody (abcam, ab1791) and an anti-p65 antibody (Santa Cruz, sc-8008) are used.

### CoIFI

In order to establish a stable human cell line, 293 F cells are transfected with plasmid pAS104.2. Monoclonal cell lines that have stably integrated the plasmid are selected by hygromycin. One of stably integrated cell lines (named clone G69: integrated plasmid copy number is ≈160, deduced from PCR analysis) is used in the CoIFI approach. Similarly, a stably transformed cell line with the plasmid lacking the TFT cassette (named clone c2-1: integrated plasmid copy number is ≈350) is established as described above and used as a negative control. The luciferase reporter assay is implemented using the ONE-Glo Luciferase assay system (Promega). For input sample preparation, G69 cells (≈1 × 10^8^ cells) are cross-linked by formaldehyde treatment (final 3%) for 30 min. Cells are re-suspended in 2-packed cell volume of pre-Lysis buffer (10 mM HEPES (7.9), 10 mM KCl, 1.5 mM MgCl_2_, 1% Triton X-100, 0.3 M Sucrose) followed by centrifugation. Cells are mixed with 3-packed cell volume of Lysis2 buffer (10 mM HEPES (7.9), 10 mM KCl, 1.5 mM MgCl_2_, 1 mM DTT, 0.1 mM EDTA, 0.5 mM EGTA, 0.6% NP40, 0.25% Triton X-100, 0.3 M Sucrose, Protease inhibitor cocktail), followed by mixing in a glass tissue homogenizer with a tight pestle. The pellet collected via centrifugation is rinsed two times with buffer PBST (PBS with 0.25% Triton X-100). The pellet is re-suspended with 3 packed cell volume of buffer PBST, supplemented with Protease inhibitor cocktail and RNaseA (final 1 mg/ml). The mixture is incubated at 37 °C for 1 hr, followed by centrifugation. The pellet is rinsed three times with buffer PBST and one time with buffer CB1.3 (15 mM Tris-Cl (7.9), 1 mM EDTA, 0.5 mM EGTA, 0.1% Sarkosyl, 0.2% SDS, Protease inhibitor cocktail). The pellet is re-suspended with 4 packed cell volume of buffer CB1.3. The mixture is treated by a Covaris S220 sonicator according to the manufacture’s instruction. Supernatant separated from insoluble fraction via centrifugation is supplemented by RNaseA (final 1 mg/ml) and exposed to streptavidin beads in order to remove proteins that bind to beads. The resultant supernatant is used as an input sample in CoIFI. Aliquot of the input (≈ 0.9 × 10^6^ cells equivalent) is mixed with 1.5 pmol of each TFO-1 and TFO-3 in buffer CB3.1 (15 mM Tris-Cl (7.9), 50 mM NaCl, 0.1 mM EDTA, 0.5 mM EGTA, 0.1% Sarkosyl, 0.2% SDS) with 10 mM MgCl_2_, and Protease inhibitor cocktail for 18 hr at 25 °C. Streptavidin T1 beads are pre-coated with 1.25 mg/ml BSA. The T1 beads are added to the mixture and agitated for 2 hr. The mixture is placed on a magnetic stand and the T1-bound fraction is processed according to the procedure described in the IDAP in *E. coli* section. The recovered supernatant is used for protein and DNA analysis after heat-induced reversal of its cross-linked state. For western blotting, the same anti-histone H3 and anti-p65 antibodies described above are used.

### Data availability

No datasets were generated or analysed during the current study.

## Electronic supplementary material

Supplementary information
