# Supplementary material for: Versatile and efficient chromatin pull-down methodology based on DNA triple helix formation
Source: Sci Rep. 2018 Apr 12;8:5925. doi: 10.1038/s41598-018-24417-9 (PMC5897567; doi:10.1038/s41598-018-24417-9)
Supplement: Supplementary file 1 — Supplementary information [file 41598_2018_24417_MOESM1_ESM.pdf]

Versatile and efficient chromatin pull-down methodology based on DNA triple helix formation

Asako Isogawa, Robert P Fuchs*$ and Shingo Fujii*

DNA Damage Tolerance CNRS, UMR7258, Marseille, F-13009, France; Inserm, U1068, CRCM, Marseille, F-13009, France; Institut Paoli-Calmettes, Marseille, F-13009, France; Aix-Marseille University, UM 105, F-13284, Marseille, France

[$]present address: Harvard Medical School, Boston MA 02115

*co-corresponding authors: robert_fuchs@hms.harvard.edu; shingo.fujii@inserm.fr

Supplementary for the section of "TFO-mediated Plasmid Capture (TmPC) *in vitro*" in the main text

Our initial attempt was to prepare biotin-conjugated plasmids either via random chemical biotinylation or by the introduction of a biotin-containing oligonucleotide. For direct biotinylation, a plasmid (pAS03: ~ 6.3 kbp) was mixed with a biotin-containing reactive aryl azide group that covalently binds to DNA upon UVA (365 nm) irradiation. Plasmid DNA was reacted with this reagent at various molar ratios leading to plasmids carrying from 1 to ≈ 300 covalently bound biotin residues per plasmid molecule (Supplementary Fig. S1a). The biotin-modified plasmids were incubated with streptavidin-coated magnetic beads in order to estimate the capture efficiency. Following various incubation times, beads are collected via a magnet. The supernatants fractions (i.e., the unbound fractions: UB) are analysed by agarose gel electrophoresis (Supplementary Figs. S1b and S1c) leading to the following conclusions: 1. *In vitro* biotinylation converts closed circular (CC) DNA to open circular (OC) and to an unidentified high molecular weight form in a dose dependent way; 2. CC plasmid is captured on beads more efficiently than OC plasmid; 3. Time dependency of capture efficiency strongly varies with the level of biotin modification. Taken together the data suggest that steric hindrance between plasmid and beads strongly limits capture efficiency. Steric hindrance appears to be partially relieved by increasing the number of biotin residues per plasmid. The down side of heavy modification is the induction of plasmid relaxation and of the possible modification of the sequence-of-interest. As our main purpose, i.e. the isolation of potentially large protein complexes assembled on DNA, may be strongly limited by steric hindrance, we decided not to pursue this approach. We also attempted to introduce a biotin-containing DNA oligonucleotide into our plasmid. We found that the length of the spacer between the plasmid and the biotin moiety is crucial for efficient capture. In contrast

to short oligonucleotides, whose capture is not limited by spacer length, circular plasmids (e.g., several kbp in size) require a spacer length in excess to 15 atoms.

Methods

IDAP in *E. coli*

DH1/pAS03 and DH1/pAS04 strains are grown to OD_{600} = ≈ 0.4 as a log phase or OD_{600} = 3 ~ 4 as a stationary phase. Cells are cross-linked by formaldehyde treatment (final 3%) for 30 min, and disrupted in buffer ECL (50 mM Tris-Cl (7.5), 10% Sucrose, 5 mM EDTA, 50 mM NaCl, 4 mg/ml lysozyme, 1 mg/ml RNaseA) for 30 min at 37°C. Buffer RW (two volume relative to the buffer ECL) is added to the mixture, followed by incubation for 30 min at 37°C. Soluble fraction is separated from the insoluble fraction by centrifugation. The recovered supernatant is passed through streptavidin magnetic beads in order to remove proteins bound to beads. The resultant supernatant is used as an input for the IDAP methodology. Aliquot of the inputs (1.9 ~ 3.3 x 10^7 cells equivalent from the log phase or 0.6 ~ 0.8 x 10^7 cells equivalent from the stationary phase; all inputs contain ≈ 400 ng plasmid equivalent) are mixed with 2 pmol of each TFO-1 and TFO-3 in buffer CB3 (15 mM Tris-Cl (7.9), 70 mM NaCl, 0.1 mM EDTA, 0.5 mM EGTA, 0.1% Sarkosyl, 0.2% SDS) with 10 mM $MgCl_2$, and Protease inhibitor cocktail (Sigma) for 18 hr at 25°C. Dynabeads MyOne Streptavidin T1 (Invitrogen) are pre-coated with 1.25 mg/ml BSA. The T1 beads are added to the mixture and agitated for 2 hr. The mixture is placed on a magnetic stand and the T1-bound fraction is collected. The collected T1 beads are washed with two times of buffer CB3 with 5 mM $MgCl_2$, two times of buffer CB4 (15 mM Tris-Cl (7.9), 70 mM NaCl, 0.1 mM EDTA, 0.5 mM EGTA, 0.1% Sarkosyl, 1% SDS) with 5 mM $MgCl_2$, two times of buffer CB3 with 1 M NaCl, and two times of buffer BR1 (10 mM Tris-Cl (7.4), 250 mM NaCl, 0.5 mM EDTA, 0.1% NP40) with 5 mM $MgCl_2$. Plasmid captured on the T1 beads is released in buffer ER1 (10 mM Tris-Cl (7.4), 250 mM NaCl, 0.5 mM EDTA, 0.1% NP40, 20 mM D-biotin) for 1 hr. The recovered supernatant is used for protein and DNA analysis after heat-induced reversal of its cross-linked state.

TFO-mediated RN01 capture

RN01 is mixed with TFO for 18 hr in buffer BR1 with 10 mM $MgCl_2$ at 25°C. C1 beads are pre-coated with 2 mg/ml BSA and 0.4 mg/ml salmon sperm DNA. The C1 beads are added to the mixture (RN01/TFO) and agitated for 30 min. The mixture is placed on a magnetic stand and the C1-bound fraction is collected. The collected C1 beads are washed under the same condition as for IDAP in *E. coli*. RN01 captured on the C1 is released in buffer ER1 for 1 hr.

Reconstitution of mono-nucleosome

Human histones are purchased from NEB, and reconstitution of nucleosome follows the manufacture's instruction. Gel shift assay for confirming status of nucleosome is implemented on a 6% DNA retardation gel, and DNAs are detected by EtBr staining.

IDAP in mono-nucleosome

Cross-linked nucleosomes (1% formaldehyde for 10 min) derived from RN01 (10 ng) are incubated with either 0.6 pmol target TFO (tTFO: a mixture of TFO-1 and TFO-3) for forming triplex or 0.6 pmol scrambled TFO (sTFO: a mixture of Scr-1 and Scr-2) as a negative control, for 18 hr in buffer BR1 with 10 mM $MgCl_2$ at 25°C. C1 beads are pre-coated with 2 mg/ml BSA and 0.4 mg/ml salmon sperm DNA. The C1 beads are added to the mixture and agitated for 30 min. The mixture is placed on a magnetic stand and the C1-bound fraction is collected. The collected C1 beads are washed under the same condition as IDAP in *E. coli*. Nucleosome captured on the C1 is released in buffer ER1 for 1 hr. For western blotting, an anti-histone H3 antibody (abcam, ab1791) is used.

a

	Reaction condition	Quantification
Sample name	Relative amount of biotin to pAS03	Number of biotin par pAS03
1	9,600	267
2	2,880	261
3	960	252
4	288	74
5	96	3
6	28.8	< 3
7	9.6	< 3
8	2.9	< 3

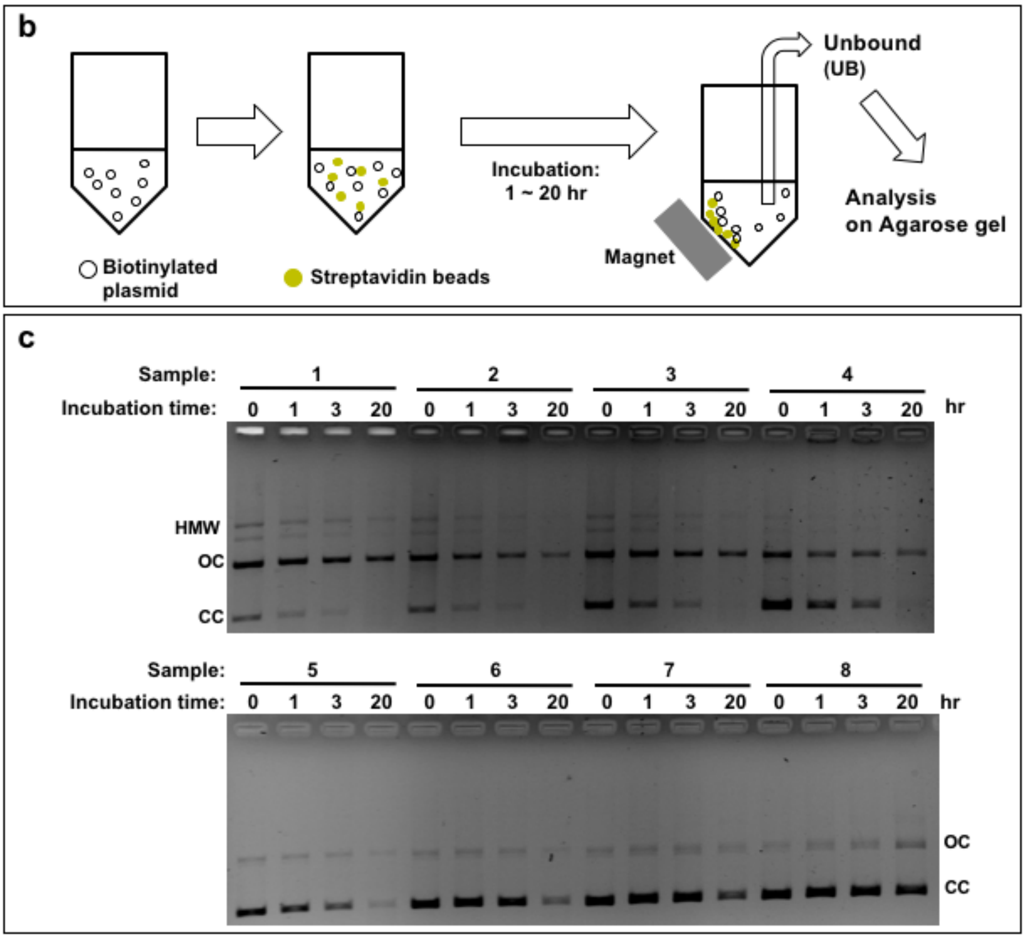

Supplementary Figure S1. Randomly biotinylated plasmid capture *in vitro*: (a) Plasmid pAS03 is directly biotinylated in using 8 different biotin/plasmid molar ratios (middle column). The number of biotin residues per plasmid molecule is determined (right column). (b) The biotinylated plasmids (500 ng) are incubated with streptavidin beads for various time periods as indicated. The unbound plasmid fractions (UB) are recovered using a magnetic stand and further analysed by agarose gel electrophoresis via EtBr staining by loading a defined volume of UB (1/10 vol). (c) Sample names (1 ~ 8) as in panel a. Initial samples (0 hr) are taken prior to mixing with beads. CC: closed circular. OC: open circular. HMW: high molecular weight.

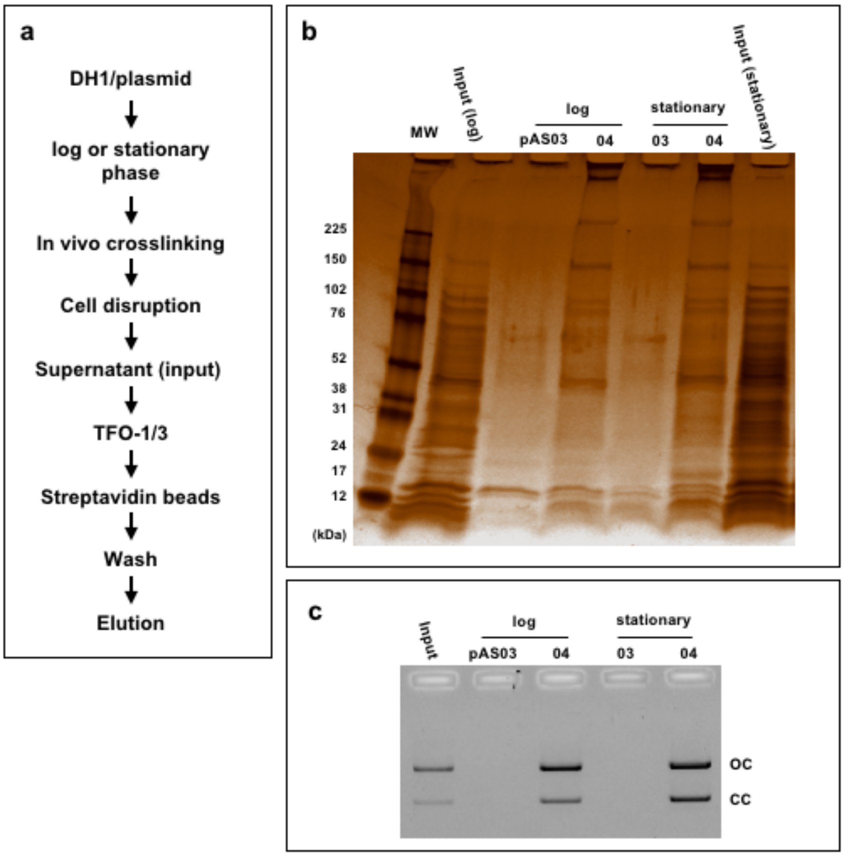

Supplementary Figure S2. Isolation of proteins bound to plasmids within *E. coli* cells: (a) Schematic view of the experimental workflow. (b) 78% of the recovered samples are reverse cross-linked and analysed by SDS-PAGE followed by silver staining. Both inputs (log and stationary phases of DH1/pAS04) are loaded ≈ 1 x 10^5 cells equivalents. MW: molecular weight marker. (c) 10% of the recovered samples are reverse cross-linked, and are analysed by agarose gel electrophoresis followed by EtBr staining. Estimation of plasmid recovery compared to input is: pAS04 in a log phase, 30%; pAS04 in a stationary phase, ≈ 40%, as quantified by Image Lab software (Bio-Rad). This experiment was independently performed four times with similar results. Input is prepared from ≈ 3 x 10^5 cross-linked cells (DH1/pAS04 in a stationary phase; all inputs showed similar DNA profile as the lane of input herein) and the amount of plasmid is ≈ 4 ng. It should be noticed that majority of ccDNA is converted to ocDNA during heat-induced reverse cross-link reaction. CC: closed circular. OC: open circular.

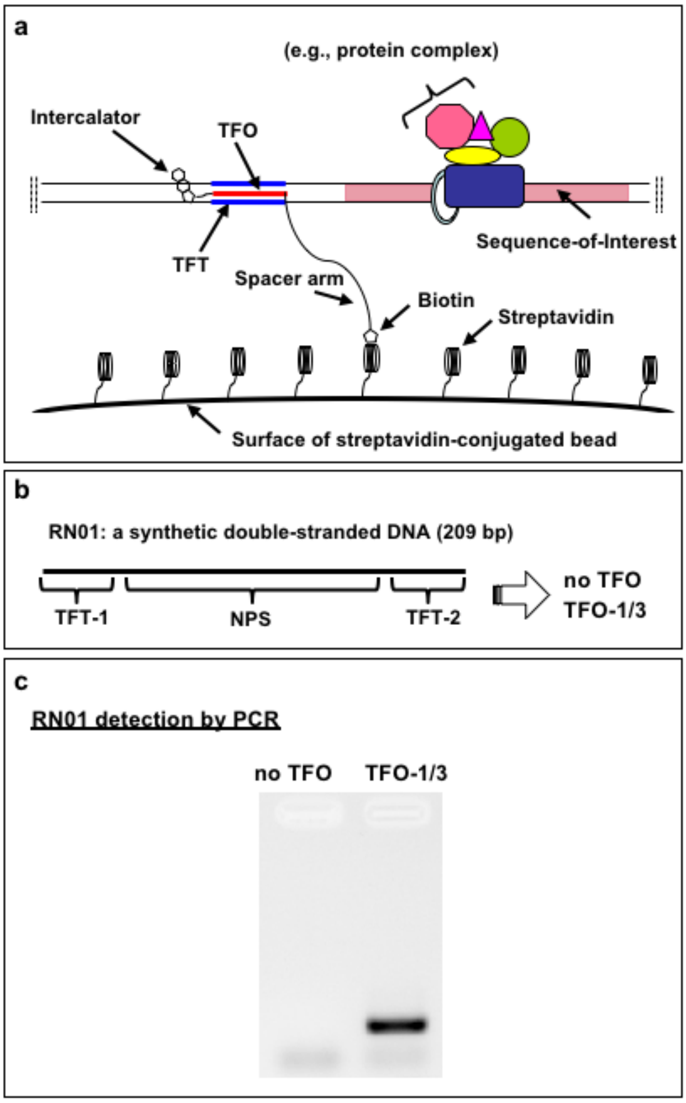

Supplementary Figure S3. TFO-mediated capture of a TFT-containing DNA sequence: (a) Strategy for isolation of protein complexes bound to a given Sequence-of-Interest. (b) RN01 is composed of a nucleosome positioning sequence (NPS) and two different TFT sites at each extremity (TFT-1/TFT-2). RN01 (10 ng) is incubated with either no TFO or 0.6 pmol TFO-1 + 0.6 pmol TFO-3. (c) Elution products from beads are amplified by PCR and analysed on agarose. PCR quantification was performed as detailed in Figure 6d. A small fraction (0.001%) of the elution products are amplified by PCR for DNA detection, quantified by Image Lab software (Bio-Rad). Experiments were independently performed three times with similar results.

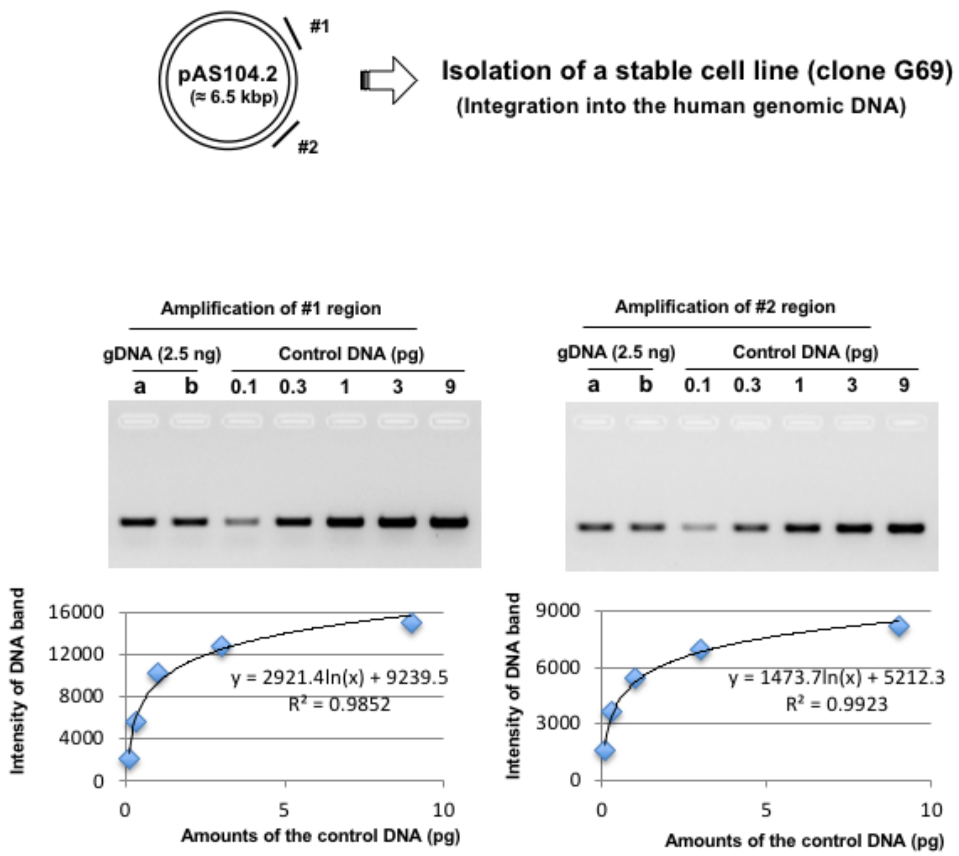

Supplementary Figure S4. Determination of the number of pAS104.2 plasmid copies integrated in the genome in clone G69: Two independent genomic DNA (gDNA) extractions, labelled a and b, are prepared from cultured G69 cells. Two plasmid regions, designated #1 and #2, are amplified by PCR from a known amount (2.5 ng) of gDNA of both a and b samples. Known amounts of pAS104.2 plasmid DNA are amplified in parallel and run on an agarose gel. Band intensities are quantified using Image Lab software (Bio-Rad) and used to construct the standard curves as shown. We determined that the average amount of plasmid DNA amplified from both samples a and b corresponds to ≈ 0.3 pg indicating that the proportion of plasmid DNA in genomic DNA represents ≈ 0.012% (i.e., 0.3 pg / 2.5 ng). The genome size of HEK293 cell (nearly triploid) [1] is estimated to be ≈ 8.9 Bbp (= a modal chromosome number of 64 x ≈ 3.2 Bbp / 23 chromosomes). The amount of plasmid DNA represents ≈ 0.012% of gDNA, thus corresponding to ≈ 1.06 Mbp. Given the size of plasmid pAS104.2 (≈ 6.5 kbp), the corresponding number of integrated copies is ≈ 160 copies per cell (1.06 Mbp / 6.5 kbp).

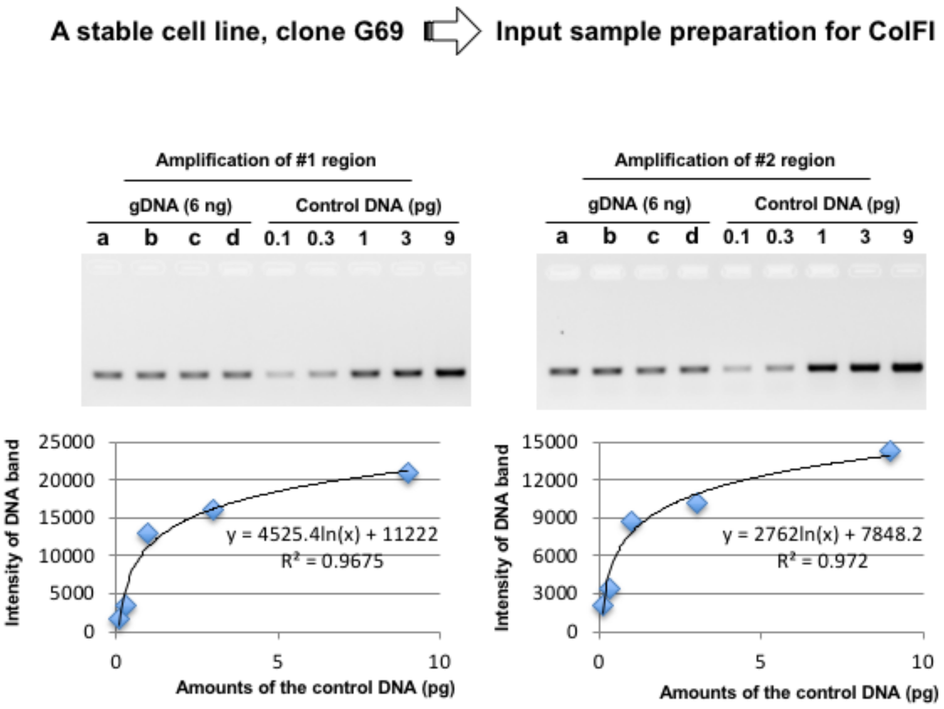

Supplementary Figure S5. Estimation of the relative proportion of target DNA segment in the input samples for ColFI: Total gDNA (clone G69) is purified from input samples (four independent samples, labelled as a ~ d) of ColFI. The same two regions in plasmid (#1 and #2 as in Supplementary Fig. S4) are amplified by PCR from 6 ng of gDNA. Known amounts of pAS104.2 plasmid DNA are amplified in parallel and run on an agarose gel. Band intensities are quantified using Image Lab software (Bio-Rad) and used to construct the standard curves as shown. We determined that the average amount of plasmid DNA amplified from samples a ~ d corresponds to ≈ 0.4 pg indicating that the proportion of plasmid DNA in genomic DNA represents ≈ 0.0067% (i.e., 0.4 pg / 6 ng). It can be noted that this value is ≈ 2 fold less than the value (≈ 0.012%) estimated during plasmid copy number determination, possibly reflecting differences in sample preparation protocols.

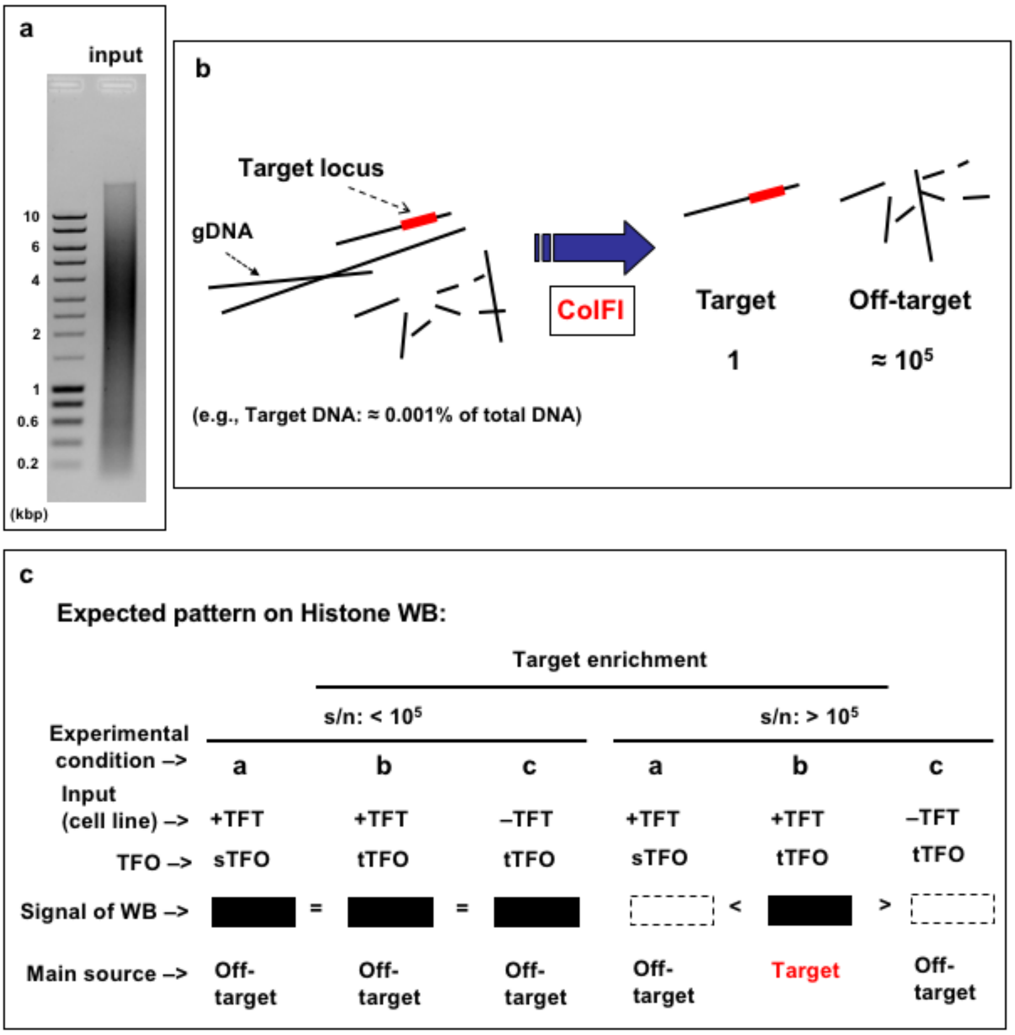

Supplementary Figure S6. Experimental design exemplifying the feasibility of CoIFI in cells: (a) A representative pattern of genomic DNA prepared from an input sample to be used in CoIFI. This input sample is prepared from G69 cell line and used in the CoIFI experiment shown in Figure 6. (b) Relative abundance of target DNA (in red) versus bulk DNA. In G69 cell line, ≈ 160 copies of plasmid construct are stably integrated. As the target DNA fragments (in red) in input are present at a ratio of one in ≈ 10^5 non-target fragments, to be successful the CoIFI procedure requires ≥ 10^5-fold enrichment. (c) Detection of histones as a way to monitor the actual enrichment during CoIFI: If an input sample is derived from G69 cells, the proportion of plasmid-derived DNA fragments to total gDNA is ≈ 0.002%. Therefore, if the signal-to-noise (s/n) ratio in CoIFI is < 10^5, the signal intensity of histone (e.g., via WB) will be similar under all three conditions a, b and c as depicted in Figure 6c. On the other hand, if the s/n ratio in CoIFI is ≥ 10^5, the signal intensity of histone (e.g., via WB) will be significantly higher under condition b compared to a and c.

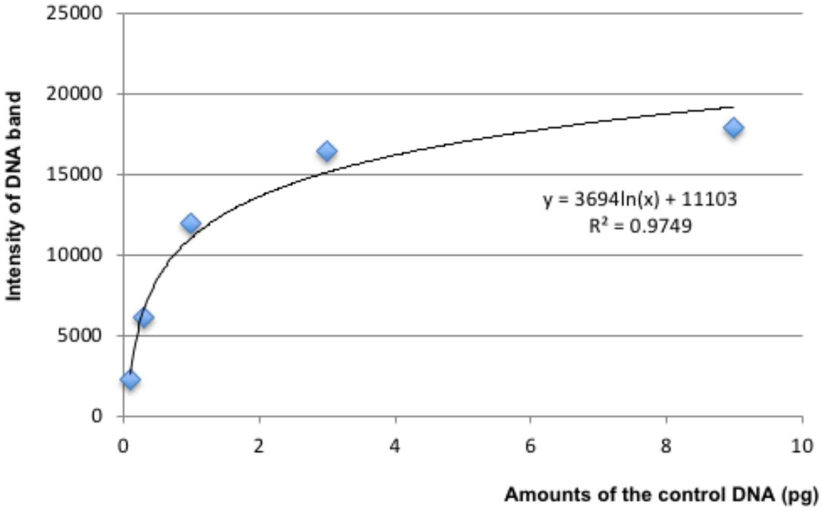

Supplementary Figure S7. Standard curve used to quantify the recovery yield of DNA segment in ColFI analysis: This curve is derived from the control DNA data shown in Figure 6d, and is used uniquely for quantification of band intensities in Figure 6d. When quantifying intensities of DNA bands of interests amplified by PCR (e.g., for test of reproducibility; in different assays), know amounts of reference DNA are also amplified in the same time and analysed in the same gel as well as data shown in Figure 6d.

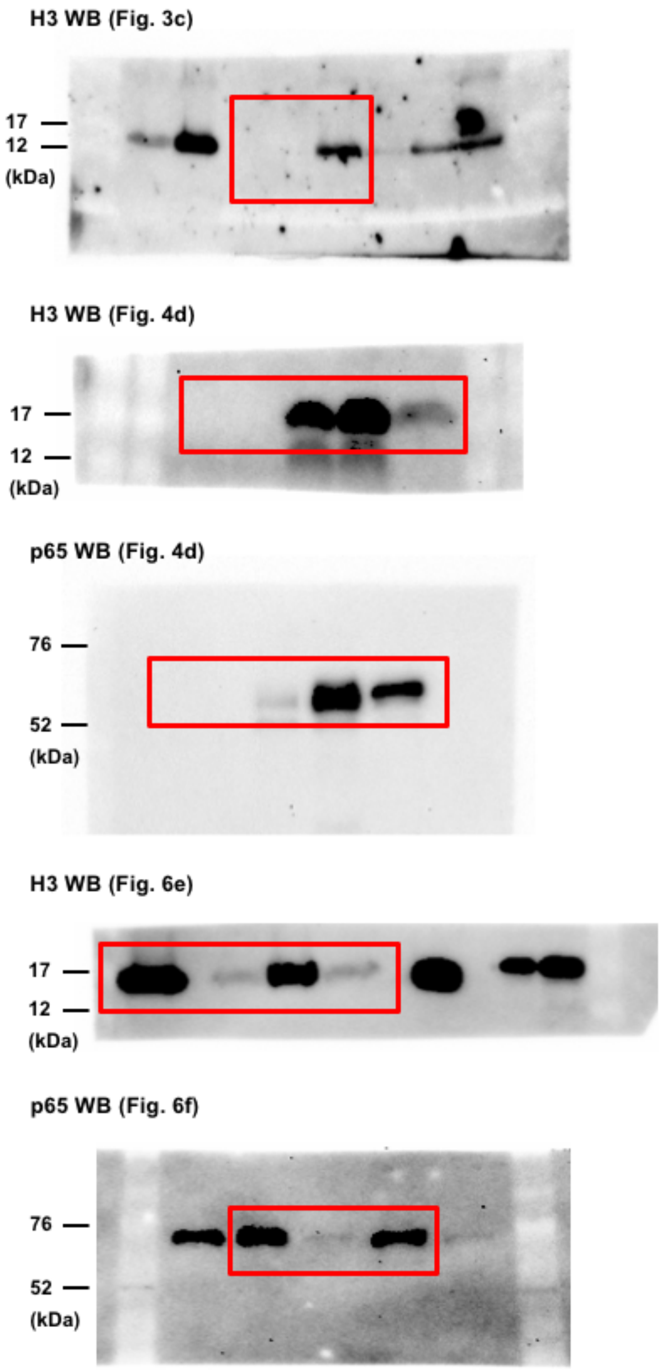

Supplementary Figure S8. Western blot data used in Figures.

References

1. Bylund, L., Kytölä, S., Lui, W. O., Larsson, C. & Weber, G. Analysis of the cytogenetic stability of the human embryonal kidney cell line 293 by cytogenetic and STR profiling approaches. *Cytogenet Genome Res* **106,** 28–32 (2004).
